# Stomach and duodenum dose–volume constraints for locally advanced pancreatic cancer patients treated in 15 fractions in combination with chemotherapy

**DOI:** 10.3389/fonc.2022.983984

**Published:** 2023-01-24

**Authors:** Sara Broggi, Paolo Passoni, Paolo Tiberio, Alessandro Cicchetti, Giovanni Mauro Cattaneo, Barbara Longobardi, Martina Mori, Michele Reni, Najla Slim, Antonella Del Vecchio, Nadia G. Di Muzio, Claudio Fiorino

**Affiliations:** ^1^ Medical Physics, San Raffaele Scientific Institute, Milano, Italy; ^2^ Radiotherapy, San Raffaele Scientific Institute, Milano, Italy; ^3^ Unit of Data Science, Department of Epidemiology and Data Science, Fondazione IRCCS Istituto Nazionale dei Tumori, Milan, Italy; ^4^ Oncology, San Raffaele Scientific Institute, Milano, Italy; ^5^ Vita-Salute San Raffaele University, Milano, Italy

**Keywords:** pancreatic cancer, radiotherapy, gastric toxicity, duodenum, dose-volume effects

## Abstract

**Purpose:**

To assess dosimetry predictors of gastric and duodenal toxicities for locally advanced pancreatic cancer (LAPC) patients treated with chemo-radiotherapy in 15 fractions.

**Methods:**

Data from 204 LAPC patients treated with induction+concurrent chemotherapy and radiotherapy (44.25 Gy in 15 fractions) were available. Forty-three patients received a simultaneous integrated boost of 48–58 Gy. Gastric/duodenal Common Terminology Criteria for Adverse Events v. 5 (CTCAEv5) Grade ≥2 toxicities were analyzed. Absolute/% duodenal and stomach dose–volume histograms (DVHs) of patients with/without toxicities were compared: the most predictive DVH points were identified, and their association with toxicity was tested in univariate and multivariate logistic regressions together with near-maximum dose (D_0.03_) and selected clinical variables.

**Results:**

Toxicity occurred in 18 patients: 3 duodenal (ulcer and duodenitis) and 10 gastric (ulcer and stomatitis); 5/18 experienced both. At univariate analysis, V44cc (duodenum: p = 0.02, OR = 1.07; stomach: p = 0.01, OR = 1.12) and D_0.03_ (p = 0.07, OR = 1.19; p = 0.008, OR = 1.12) were found to be the most predictive parameters. Stomach/duodenum V44Gy and stomach D_0.03_ were confirmed at multivariate analysis and found to be sufficiently robust at internal, bootstrap-based validation; the results regarding duodenum D_0.03_ were less robust. No clinical variables or %DVH was significantly associated with toxicity. The best duodenum cutoff values were V44Gy < 9.1 cc (and D_0.03_ < 47.6 Gy); concerning the stomach, they were V44Gy < 2 cc and D_0.03_ < 45 Gy. The identified predictors showed a high negative predictive value (>94%).

**Conclusion:**

In a large cohort treated with hypofractionated radiotherapy for LAPC, the risk of duodenal/gastric toxicities was associated with duodenum/stomach DVH. Constraining duodenum V44Gy < 9.1 cc, stomach V44Gy < 2 cc, and stomach D_0.03_ < 45 Gy should keep the toxicity rate at approximately or below 5%. The association with duodenum D_0.03_ was not sufficiently robust due to the limited number of events, although results suggest that a limit of 45–46 Gy should be safe.

## 1 Introduction

Pancreatic cancer is one of the leading causes of cancer-related death in Europe and North America (1). Most patients are still unresectable at diagnosis, with the large majority presenting at locally advanced stage or metastatic (2, 3). Despite some advancements, the prognosis of locally advanced pancreatic cancer (LAPC) remains poor, with median overall survival around approximately 12–15 months (2–7). A major cause for this unsatisfactory result lies in the prevalent metastatic progression; however, the role of improving local control through local therapy intensification has been underlined suggesting the exploration of “safe” ways to escalate the dose to the tumor (8–11). There is, in fact, some mounting evidence that a fraction not negligible of patients could benefit in terms of overall survival from improved local control, although this is not yet precisely quantified (9–14).

The technological developments in radiotherapy imaging and precision delivery (15) pushed researchers in investigating dose-escalated protocols, mostly using image-guided radiation therapy (IGRT) aiming to reduce planning target volumes (PTVs) around more precisely defined target volumes (clinical target volume (CTV) and internal target volume (ITV)). A relevant issue concerns the proximity of organs at risk (OARs), primarily the stomach and duodenum, whose sparing is crucial to avoid severe toxicities. The way these OARs are spared heavily influences the ability to deliver sufficiently high doses to the tumor. The small and uncertain benefit of stereotactic body radiotherapy (SBRT) delivered in 1–5 fractions (14, 16–21) is likely to depend on this issue, despite the use of advanced technology to reduce the impact of inter- and intra-fraction motion (15, 22–25); new developments in MRI-Linacs (23) promise to improve the picture, but the experience is still too early, and the spread of these machines is not expected to move rapidly.

In the last years, the interest toward moderate hypofractionation, also combined with concomitant dose escalation on portions of PTV (8, 9, 11, 12, 22), is increasing, suggesting that schedules with a number of fractions equal to 15 or around this value may represent an optimal window to deliver sufficiently high dose by keeping low the rate of gastric/duodenal toxicities (9, 12, 26). Moreover, the relatively large number of fractions intrinsically reduces the impact of unusual anatomy deformation in single fractions, compared to SBRT (25). However, there is still an evident lack of knowledge of dose–volume effects for these organs under these fractionations, with few published studies regarding relatively small populations (22, 26–29). This lack may reflect a limitation in exploiting the potential of dose escalation, due to a likely “over-safe” approach in sparing OARs. Our institute was among the first ones to adopt a moderate hypofractionation approach using 15 fractions since 2004, including concomitant dose escalation in sub-volumes within a Phase I trial (8).

In a pilot investigation on the first 61 patients, Cattaneo et al. (27) found a significant association between stomach/duodenum dose–volume histograms (DVHs) and gastric/duodenal toxicities.

The aim of the current study was to update the previous results on a much larger population of 204 LAPC patients treated in a quite homogeneous way, delivering 44.25 Gy in 15 fractions using helical tomotherapy (HT); based on these results, rational constraints were derived even in the light of a renewed interest toward the promising field of dose-escalated radiotherapy delivered in 15 fractions (30).

## 2 Materials and methods

### 2.1 Eligibility criteria

The current analysis refers to patients with histologically confirmed LAPC treated according to an institutional protocol (see below), from 2004 to 2019 at San Raffaele Institute in Milan. Patients excluded from surgery because judged unresectable were submitted to induction chemotherapy; schedules changed over time, consisting in most patients of four to six cycles of four-drug combinations: cisplatin, epirubicin, 5-fluorouracil or capecitabine, and gemcitabine (acronyms PEFG and PEXG). After completing induction chemotherapy, patients were restaged and discussed at multidisciplinary team meetings. Considered for radio-chemotherapy (RCT) were 1) patients in stage III still deemed not resectable due to vascular encasement, including patients with local progression after chemotherapy or with increased CA 19.9 compared to the nadir value reached during chemotherapy, and 2) patients in stage IV with complete response of metastases stable over a period of at least 4 months after the end of induction chemotherapy. Among those treated with RCT, the criteria for inclusion in the current analysis were as follows: a) histological diagnosis of pancreatic adenocarcinoma, b) radiotherapy delivered with HT, c) Karnofsky performance status scale >70, d) age > 18 years, and e) availability of complete treatment planning data. In total, the resulting cohort included 204 patients.

### 2.2 Treatment characteristics

RCT was generally planned 2–4 weeks after the completion of induction chemotherapy. It consisted of the delivery of 44.25 Gy in 15 fractions, concomitant to capecitabine, 1,250 mg/day weekend included, for 3 weeks. Details of radiotherapy procedures are reported elsewhere (8, 27, 31). In short, patients were immobilized and underwent simulation contrast-enhanced CT and FDG-PET/CT. Primary tumors and enlarged lymph nodes visible on the contrast-enhanced CT images or 4D-CT images were defined as gross tumor volume (GTV). When a standard CT was performed, ITV was defined as GTV with a margin of 0.5 cm in anterior–posterior and left directions, and of 1.0 cm in cranial–caudal direction. In the case of 4D-CT, GTV was contoured on at least four phases, and an ITV was obtained by the union of four GTVs. The PET-positive volume and the biological target volume (BTV), when available, were merged with ITV to create the ITV/BTV. A further margin of 0.5 cm in all directions was added to ITV/BTV to create PTV. The stomach, duodenum, liver, kidneys, and spinal cord were contoured as OARs. Constraints for the stomach were V40 < 18 cc and V30 < 23 cc; constraints for duodenum were V45 < 1 cc, V40 < 15 cc, and V30 < 35 cc. The dose prescription to the overlap between PTV and the stomach/duodenum was 44.25, 43.25, and 42.25 Gy, when the overlap volume was <14, 30, and 50 cc, respectively. In case of overlap >50 cc or dose constraints were not recognized, the dose to the whole PTV was reduced to 40 Gy. All treatment plans were generated using the HT planning system. With regard to PTVs, the goal was to deliver ≥95% of the prescribed dose to ≥95% of the volume while keeping the dose as homogenous as possible. During the optimization process, the planner had to reduce the volume of irradiated stomach+duodenum as much as possible while maintaining tumor coverage as the highest priority.

All treatment plans were generated using tomotherapy inverse planning software, using the same convolution/superposition dose calculation algorithm.

Fifteen patients received an additional boost to a sub-volume PTV2 obtained from the infiltrating vessels with doses ranging from 48 to 58 Gy. Details are described elsewhere (8). In addition, 28 patients with favorable tumor dimensions or tumor anatomic sites with respect to dose constraints received a simultaneous integrated boost of 48 Gy to BTV. Patients with simultaneous integrated boost (SIB) to infiltrating vessels were included in a Phase I trial. The protocol was approved by our Institutional Ethical Committee. Once it was confirmed that the delivery of 44.25 Gy in 15 fractions was feasible, the Phase I protocol was amended, and a subsequent observational perspective trial was approved for the remaining patients; all patients provided written informed consent. For all patients, a megavoltage CT was performed before each fraction and co-registered with the planning CT by means of automatic matching on bony anatomy, followed by manual refinement based on daily patient anatomy. The physician further checked and corrected the patient setup by means of direct visualization of other anatomical details. Of note, patients were carefully instructed to have empty stomach both at planning CT and during treatment delivery.

### 2.3 Toxicity scoring, end-point definition, and DVH recovery

Patients were examined once a week during treatment by radiation and medical oncologists. Adverse events were classified as acute or late toxicity when taking place during the treatment and within 3 months after RCT completion or 3 months after, respectively. Toxicity was scored by the National Cancer Institute Common Terminology Criteria for Adverse Events (CTCAE). For the current study, gastric and duodenal CTCAEv5 Grade ≥2 toxicities were considered. Due to the limited number of events, acute and late events were considered together. DVHs (absolute and %) of the stomach and duodenum as previously contoured by the treating physician were recovered. Percentage and absolute stomach and duodenum volumes receiving more than XGy, with X ranging at 1–60 Gy, were extracted with a 1-Gy step.

### 2.4 Quantifying the relationship between DVH and toxicity

Average absolute/% DVHs of the stomach/duodenum for patients with/without toxicity were compared through a two-sided *t*-test, according to a previously applied approach (32–34): the DVH regions corresponding to the lowest p-values were considered as candidate values to be tested in a logistic regression analysis as potential dosimetry predictors. The best cutoff values discriminating patients with/without toxicity were also assessed by receiver operating characteristic (ROC) curves, according to the DeLong method, using Youden’s index (35), aiming to define optimal constraints. Univariable logistic regression (UVA) was performed to assess the correlation between the considered end-points and the selected dosimetric parameters; selected clinical variables were also tested (gender, age, stage (III *vs.* IV), drugs used as induction chemotherapy (one *vs.* multiple drugs), number of induction chemotherapy cycles (≥6 *vs.* <6), Karnofsky performance status, and patient’s weight). Variables with p-value <0.1 at UVA and without cross-correlations (Pearson’s or Spearman’s coefficient, in the range of −0.25 to 0.25) were entered into a backward stepwise multivariable logistic regression (MVA). The goodness of fit was assessed by the Hosmer–Lemeshow test (H&L). Analyses were performed with the MedCalc software (v.19.0.4, MedCalc Software bvba, Ostend, Belgium) and the R software version 3.2.4 (^©^The R Foundation for Statistical Computing, Vienna, Austria). Due to the limited number of events, an internal validation procedure was performed using a dedicated script in Matlab by repeating the regression fit for the major dosimetry predictors on 500 data sets obtained by bootstrapping the original cohort. Median and inter-quartile ranges of p-values, odd ratios (ORs), and AUC values obtained by the procedure were reported and compared with the results obtained on the original cohort, as a measure of the results’ robustness.

## 3 Results

### 3.1 Patient characteristics

The main characteristics of patients were summarized in **Table 1**: 184 patients were in stage III, and 20 were in stage IV. Most patients (n = 177) received a combination of at least two drugs as induction chemotherapy. The median number of cycles was 6 (range, 2–13), and 108 patients received ≥6 cycles. Most patients (n = 135) were treated with a dose of 44.25 Gy; 26 patients with 40 Gy and 43 patients received a SIB of up to 48–58 Gy. The median follow-up was 18 months, and the median overall survival was 19.5 months (from the start of induction chemotherapy). Eighteen patients (8.8%) had gastric and/or duodenal mucosal damage CTCAEv5 Grade ≥2 toxicities (5 acute and 13 late): 3 patients only duodenal, 10 patients only gastric, and 5 patients both duodenal and gastric damage. Of 18 patients, 10 were treated with SIB. The median time to toxicity was 5 months (range, 1–10) from the end of RCT.

**Table 1:** Main characteristics of considered patients.

All patients: 204	
Age	65 (40–86)
Gender	Male: 92 Female: 112
KPS	90 (70–100)
Stage	III: 184 IV: 20
Weight	66.5 (41–104)
Location of primary tumor	Head (+uncinato): 103 Head/isthmus: 13 Head/body: 10 Isthmus: 10 Body: 30 Body/isthmus: 16 Body/tail: 20 Tail: 2
Induction chemotherapy	One drug: 27 Multiple drugs: 177
Number of chemotherapy cycles	≥6 cycles: 108 <6 cycles: 83
Doses	40 Gy: 26 44.25 Gy: 135 48–58 Gy (infiltrating vessels):15 48–50 Gy (BTV SIB): 28

KPS, Karnofsky Performance Status; BTV, biological target volume; SIB, simultaneous integrated boost.

### 3.2 Assessing dosimetry predictors

**Figure 1:**
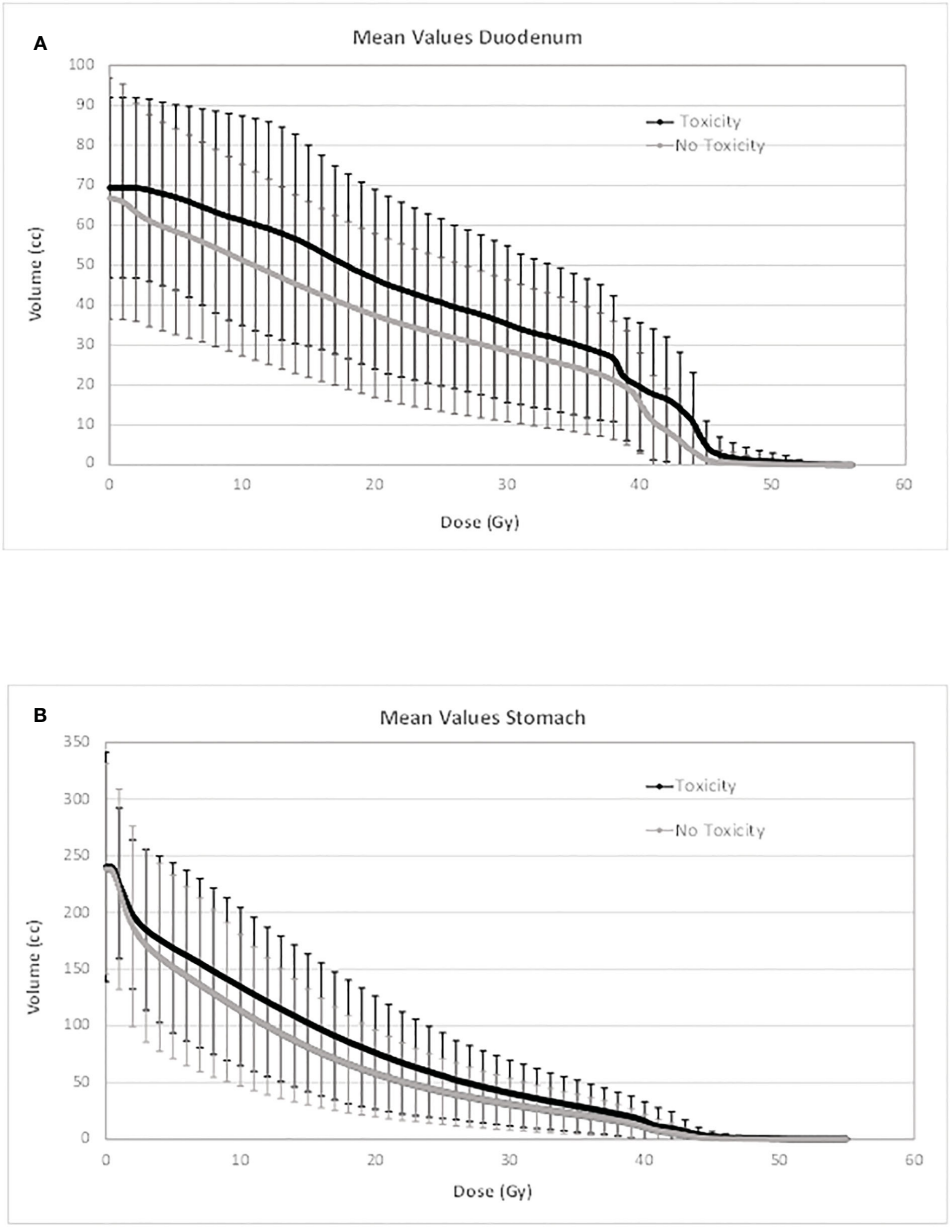
Average absolute DVH for duodenum **(A)** and stomach **(B)** for patients with and without toxicities. DVH, dose–volume histogram.

**Figure 2:**
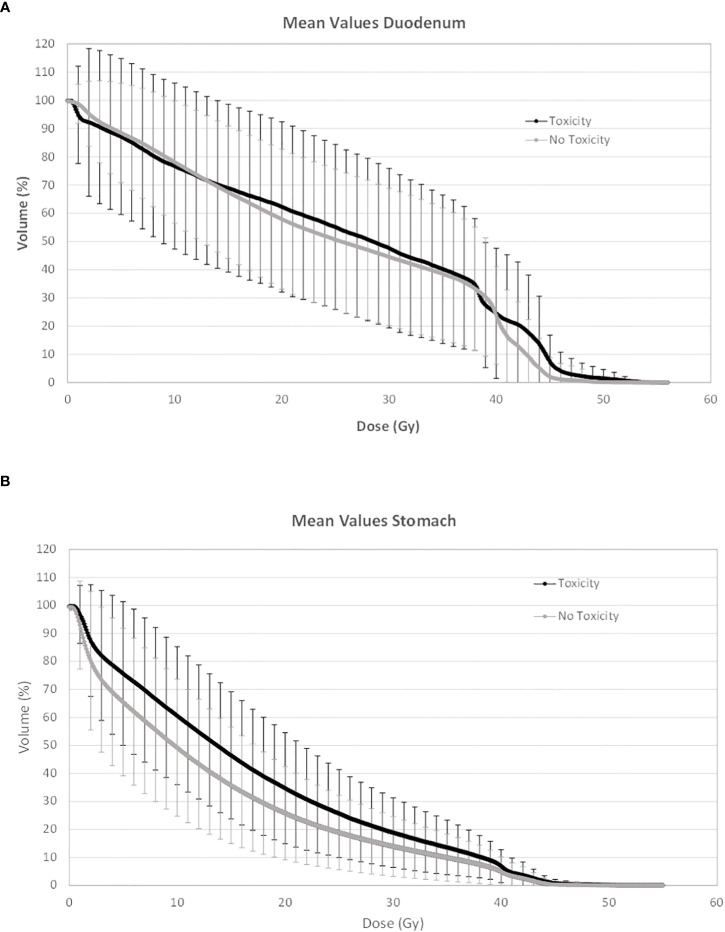
Average percentage DVH for duodenum **(A)** and stomach **(B)** for patients with and without toxicities. DVH, dose–volume histogram.

In **Figures 1** and **2**, the average absolute/percentage DVHs for the duodenum and stomach for patients with and without toxicities were reported. In **Figures S1 and S2 (Supplementary Material)**, the *t*-test graphs for absolute and percentage DVHs were shown. For both duodenum and stomach absolute DVHs, the absolute volumes (in cc) that receive 15 Gy (V15cc) and 44 Gy (V44cc) were selected through a two-sided *t*-test as the most promising discriminating DVH parameters. For percentage DVHs, V20 (%) and V44 (%) were found to be the most discriminating DVH parameters for duodenum; V15 (%), V20 (%), and V44 (%) were found to be the most discriminating DVH parameters for the stomach.

At univariate logistic regression analysis, the previously selected DVH parameters and Dmax (D_0.03_) were tested as potential dosimetry predictors of gastric and duodenal toxicity. Duodenum V44Gy(cc) (p = 0.02; OR = 1.07) was found as the only significative predictive parameter for duodenal toxicity; stomach D_0.03_ (p = 0.008; OR = 1.23) and V44Gy(cc) (p = 0.01; OR = 1.12) were found as the most predictive parameters for gastric toxicity. A borderline significance was found for duodenum D_0.03_ (p = 0.07; OR = 1.19) for duodenal toxicity. None of the percentage dosimetric parameters selected through a two-sided *t*-test were found predictive, neither for duodenal nor gastric toxicity (**Table S1, Supplementary Material**). Based on a ROC analysis, duodenum V44Gy > 9.1 cc was found to be the best cutoff value for duodenal toxicity with a negative predictive value (NPV) of 97.6%; although near to the significance, D_0.03_ > 47.6 Gy was found as the best cutoff value for duodenal toxicity. For the stomach, D_0.03_ > 45 Gy and V44Gy > 2 cc were found as the best cutoff values for gastric toxicity, with NPVs equal to 95.8% and 95.4%, respectively (**Figure S3, Supplementary Material**).

The crude rate of duodenal toxicity was 4/176 (2.3%) *vs.* 4/28 (14.3%) (p = 0.012) if duodenum V44Gy <9.1 or ≥9.1 cc, respectively. The crude rate of gastric toxicity was 6/145 (4.1%) *vs.* 9/58 (15.5%) (p = 0.012) if stomach D_0.03_ <45 or ≥45 Gy, and 7/158 (4.4%) *vs.* 8/45 (17.8%) (p = 0.007) if V44Gy <2 or ≥2 cc. Of note, the incidence of Grade ≥2 mucosal damage was 10/43 (23.3%) for patients treated with doses 48–58 Gy *vs.* 8/161 (5.0%) for patients treated with 44.25 Gy or less (p = 0.0002).

The internal validation procedure was applied to V44Gy and D_0.03_ of both the duodenum and stomach, respectively, for duodenal and gastric toxicity end-points. Results, reported in the Supplementary Material Results, confirmed sufficiently high robustness of the found associations, confirming duodenum V44Gy and stomach D_0.03_ as the most robust predictors for duodenal and gastric toxicities, respectively.

### 3.3 Multivariable analysis

**Figure 3:**
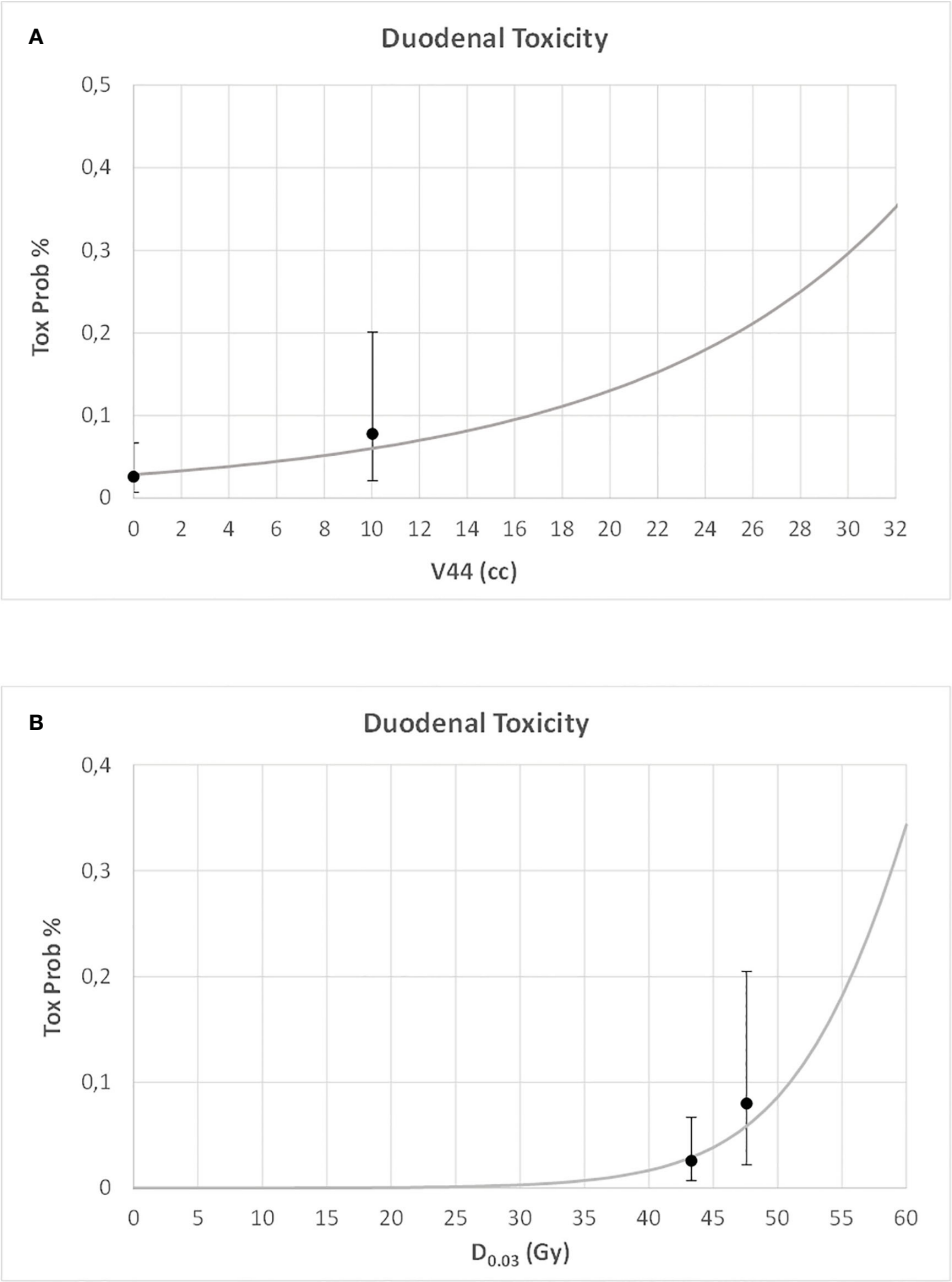
Risk of duodenal toxicity against duodenum V44 **(A)** and maximum dose **(B)** (D0.03), together with the true rates and their standard deviations.

**Figure 4:**
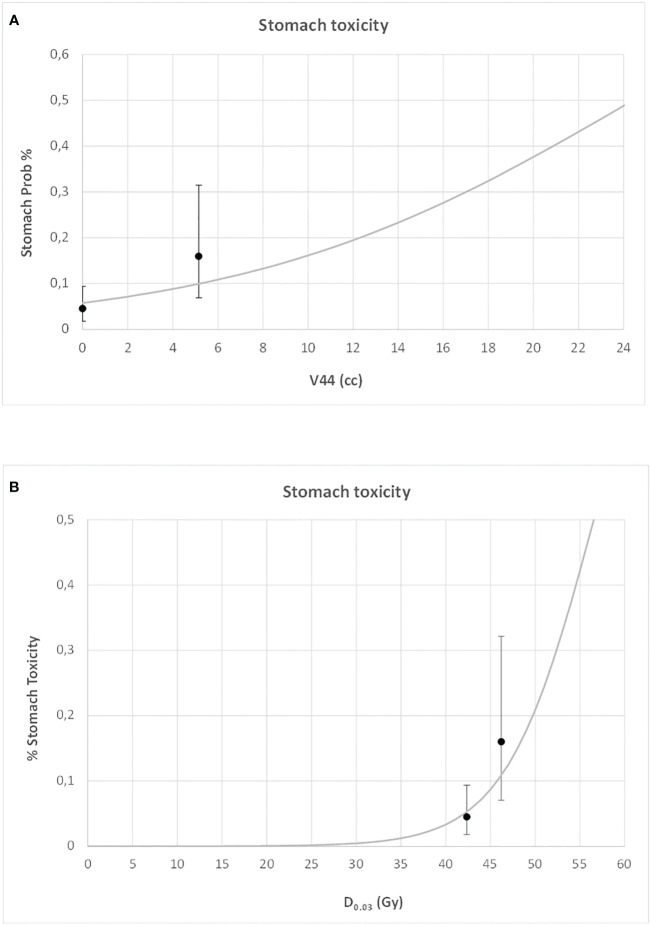
Risk of gastric toxicity against stomach V44 **(A)** and maximum dose **(B)** (D0.03), together with the true rates and their standard deviations.

For both duodenal and gastric toxicities, no significant correlations were found with clinical variables at univariable analysis (**Table S2, Supplementary Material**). In a backward stepwise logistic multivariate analysis, considering the previously selected dosimetry predictors and the clinical variables, only stomach D_0.03_ (Gy) and duodenum V44Gy were confirmed at multivariate analysis for gastric and duodenal toxicities, respectively (H&L > 0.05). In **Figures 3** and **4**, the risk of duodenal and gastric toxicities against duodenum/stomach V44Gy/D_0.03_ is plotted together with the true rates.

## 4 Discussion

Knowledge concerning quantitative relationships between dose or dose–volume metrics and the risk of toxicity for the stomach and duodenum after radiotherapy is still lacking. However, a recently accomplished review (36) showed substantial improvement in the last few years. More quantitative information was reported in the contexts of conventionally fractionated radiotherapy (i.e., 1.8–2.0 Gy/fr) and SBRT (delivered in 1–5 fractions). Concerning conventional fractionation, most studies were consistent in suggesting a prevalent dose effect when considering moderate/severe duodenal and gastric toxicities, with the risk rapidly increasing for prescribed doses above 55–60 Gy and fractions of duodenum/stomach receiving more than 35–55 Gy above few %/few to tens of cubic centimeters (28, 36–40): similar findings were suggested for mild hypofractionation (2.15–2.25 Gy/fr) in a cohort of 105 patients treated with intensity-modulated radiotherapy (IMRT) for esophageal cancer at 60.2 Gy (29). Regarding SBRT, safe constraints for stomach and duodenum were suggested for one/three/five fractions, with quite consistent recent updates based on patient data, mostly for the duodenum with the 5-fraction scheme, as reviewed by Cattaneo and Marrazzo (36). It is worth mentioning that a quite large variability in terms of treated site and variable usage of chemotherapy may partly jeopardize the generalizability of the reported results.

Approaches using moderate hypofractionation have been suggested by us and other groups as a promising way to deliver higher BED to LAPC patients to reduce the risks of delivering a too-high dose to the adjacent stomach and/or duodenum. Different from SBRT, the smaller dosimetry gap between the prescribed dose and the constrained dose to these organs could limit severe underdosing to fractions of GTV in a large part of patients. The choice of a number of fractions of approximately 15 seems to be a good compromise, and several groups recently reported promising results using this fractionation scheme (8–11, 22, 30). However, the lack of knowledge concerning the dose–volume relationships, in this case, is pushing researchers to apply strict dose limits to the stomach/duodenum (11, 22), resulting in very mild toxicity profiles (10, 22, 30). This point suggests that it is likely that a larger window exists to be explored once dose and/or dose/volume limits are better assessed, with the potential to further reinforce the impact of dose escalation on local control. In this scenario, our experience with the 15-fraction scheme with limited dose escalation to sub-volumes of PTV (in a fraction of patients) may help in better assessing refined constraints. As a matter of fact, our results, representing the largest series analyzed with this aim to our knowledge, confirm that the shape of the DVH tail of the stomach and duodenum is associated with the risk of moderate/severe toxicities. Regarding the stomach, D_0.03_ < 45 Gy and V44Gy < 2 cc may be suggested as sufficiently safe; however, V44Gy < 9 cc was found to be a robust constraint for duodenum, while the association with D_0.03_ was found to be of borderline significance: despite that the best cutoff value suggested a threshold of 47.6 Gy, the lack of robustness seen at internal validation suggests a safer limit in the range 45–46 Gy as reasonable. These values are slightly higher than the ones applied in recent dose escalation trials for duodenum and similar for the stomach (22), suggesting a likely larger potential to be exploited by dose escalation. In **Table 2** a summary of recent studies dealing with dose–volume relationships of the stomach and duodenum with moderate hypofractionation is shown. The different planning techniques, toxicity definitions, and the number of fractions make the comparison among these studies quite difficult. However, it is important to underline that the current study is the largest in terms of the number of treated patients. Compared to our previous preliminary analysis on 61 patients, only the result regarding Dmax of the duodenum was substantially confirmed, while only %DVH was analyzed in those studies. Current analysis revealed that, as expected, the absolute DVH (in cc) resulted in a better association with toxicity compared to %DVH. Huang et al. (41) also used %DVH, finding an association for duodenal toxicity: however, their study included almost only 3D conformal radiotherapy (CRT) patients, resulting in large fractions of duodenum included in the high dose regions, which is quite far from the actually delivered dose distributions. The results reported by Liu et al. (29) regarding duodenum are quite consistent with our results despite the limited number of patients: no relationships were found for the stomach.

**Table 2:** Summary of the literature for moderate hypofractionation.

Stomach									
Study		No.	End-point	No. fr	Dose Gy	Cht	Dmax Gy	DVH	Notes
Cattaneo (26)		61	≥2 CTCAE v.3	15	44.25	Ind+conc	–	V20<31%	SIB to infiltrating vessels PTV in 23/61 pts (48–55 Gy; overlap with stomach, 44.25 Gy)
Shinoto (25)		58	1 year ≥2 ulcer	12	55.2	Ind+conc	–	V10 < 102 cc V20 < 24 cc V30 < 6 cc	RBE-weighted dose (carbon ions). D2cc of GI tract constrained to 46 Gy
Liu (28)		68	≥2 CTCAE v.4	15 or 20	50/60 or 70/80	Conc 29/68	–	–	High rate of tox (late, 26%); no separation between 15 and 20 fractions in the analysis: stomach constraints: D_max_ < 60; D1 < 55; D3 < 50; D5 < 45; D10 < 40
Koay (21)		–	n.a.	15	37.5	Ind+conc	45	–	Suggested, based on experience. SIB to PTV derived from GTV and 4D CT to 67.5 Gy
Current study		204	≥2 CTCAE v.5	15	44.25	Ind+conc	45 D_0.03cc_	V44 < 9.1 cc	SIB to infiltrating vessels PTV/GTV in 43/204 pts (48–58 Gy); overlap with stomach constrained to 40–44.25 Gy depending on volume
Duodenum									
Study	No.		End-point	No. fr	Dose Gy	Cht	Dmax	DVH	Notes
Cattaneo (26)	61		≥2 CTCAE v.3	15	44.25	Ind+conc	–	V40 < 16% V45 < 2.6%	SIB to infiltrating vessels PTV in 23/61 pts (48–55 Gy; overlap with stomach, 44.25 Gy)
Huang (40)	46		≥2 CTCAE v.3 ≥3 CTCAE v.3	15	36	Conc		V25 < 45% V35 < 20%	High rate (37% 1 year). 87% pts treated with 3D RCT ≥3 CTCAE v.3 analyzed only for 28 pts without erlotinib
Liu (28)	68		≥2 CTCAE v.4	15 or 20	50/60 or 70/80	Conc 29/68	–	V45 < 0.5cc	High rate of tox (late, 26%); no separation between 15 and 20 fractions in the analysis: stomach constraints: D_max_ < 55; D1 < 50; D3 < 45; D5 < 40; D10 < 35
Koay (21)	–		n.a.	15	37.5	Ind+conc	45	–	Suggested, based on experience. SIB to PTV derived from GTV and 4D CT to 67.5 Gy
Current study	204		≥2 CTCAE v.5	15	44.25	Ind+conc	45–46* D_0.03cc_	V44 < 9.1 cc	SIB to infiltrating vessels PTV/GTV in 43/204 pts (48-58 Gy); overlap with stomach constrained to 40–44.25 Gy depending on volume

CTCAE, Common Terminology Criteria for Adverse Events; SIB, simultaneous integrated boost; BTV, biological target volume; RBE, relative biological effectiveness; GI, gastrointestinal; PTV, planning target volume; GTV, gross tumor volume.

*Best cutoff value, 47.6 Gy; suggested 45–46 Gy as safer due to limited number of events.

A major limitation of the current analysis consists in the consideration of both acute and late toxicities in a unique end-point. This was necessary in order to consider a sufficient number of events, being the late events only 11/204 (5.5%): of note, the longer time between the end of treatment and toxicity was 10 months, suggesting that the occurrence of late toxicities is in continuity with more acute events. The low number of toxicities confirmed that the irradiation to a total dose of approximately 44–45 Gy is safe, as demonstrated by the much higher rate of toxicities in the sub-groups of patients treated with SIB. However, it is important to underline that the current cohort represents the largest group analyzed with this intent and that the suggested constraints should be considered as a robust basis for future “safe” dose-escalation trials to be prospectively confirmed.

## 5 Conclusions

Current analysis suggests that constraining Dmax (D_0.03_) and V44Gy of the stomach and duodenum within 45 and 45–46 Gy and a few cubic centimeters (2 cc for the stomach and 9 cc for the duodenum, respectively) should be effective in maintaining duodenal and gastric toxicities at approximately or below 5% when delivering radiotherapy in 15 fractions to LAPC patients, combined with chemotherapy. These values are consistent with the possibility of substantially escalating the dose to the tumor without relevant risks of toxicity in a likely large fraction of patients, corroborating the promise of significantly increasing local control without any relevant increase of toxicity in future trials.

## Data availability statement

The datasets analyzed during the current study is not available due to restrictions inherent to EC approval. Requests to access the datasets should be directed to fiorino.claudio@hsr.it.

## Ethics statement

The studies involving human participants were reviewed and approved by San Raffaele Hospital. The patients/participants provided their written informed consent to participate in this study.

## Author contributions

CF, SB, and PP designed the study; PP, NS, and SB took care of data base building and data extraction; PT, AC, BL, and SB extracted and analyzed 3D planning data; SB, CF, and MM performed data and statistical analyses; SB, CF, and PP interpreted results; SB, CF, and PP wrote the main text; CF, MR, GC, AV, and NM supervised the discussion; All authors read and edited the text. All authors contributed to the article and approved the submitted version.
