# Supplementary material for: Stomach and duodenum dose–volume constraints for locally advanced pancreatic cancer patients treated in 15 fractions in combination with chemotherapy
Source: Front Oncol. 2023 Jan 24;12:983984. doi: 10.3389/fonc.2022.983984 (PMC9902495; doi:10.3389/fonc.2022.983984)
Supplement: Supplementary file 1 [file DataSheet_1.docx]

**Supplementary material**


Figure 1Sa)

Figure 1Sb)

**Figure 1S: Two-sided t-test for duodenum (1a) and stomach (1b) absolute DVHs.**


Figure 2Sa)

Figure 2Sb)

**Figure 2S: Two-sided t-test for duodenum (2a) and stomach (2b) percentage DVHs.**

**Figure 3S: ROC analysis curve**

| **Duodenum Toxicity** | | | |
| --- | --- | --- | --- |
| ***Variable*** | ***p*** | ***OR*** | ***95% CI*** |
| **D_0.03_ (Gy)** | 0.07 | 1.19 | 0.99 – 1.43 |
| **V15 (cc)** | 0.56 | 1.01 | 0.98 – 1.03 |
| **V44 (cc)** | 0.02 | 1.07 | 1.01 – 1.14 |
| **V20(%)** | 0.96 | 1.00 | 0.97 – 1.03 |
| **V44(%)** | 0.13 | 1.03 | 0.99 -1.08 |
| **Stomach Toxicity** | | | |
| **D_0.03_ (Gy)** | 0.01 | 1.23 | 1.05 - 1.43 |
| **V15 (cc)** | 0.19 | 0.99 | 0.97 - 1.01 |
| **V44 (cc)** | 0.01 | 1.12 | 1.02 - 1.23 |
| **V15 (%)** | 0.30 | 1.01 | 0.99 - 1.04 |
| **V20 (%)** | 0.16 | 1.02 | 0.99 - 1.05 |
| **V44 (%)** | 0.11 | 1.17 | 0.96 - 1.41 |

**Table 1S**: Univariate logistic regression including dosimetric variables selected through two-sided t-test, for duodenum and gastric toxicity.

**Results of internal validation**

We report the values of p-value, AUC of the ROC curve and OR for the four parameters selected in the study as mostly associated to duodenum and stomach toxicities: the original values were compared against the median values of 500 regression fits obtained from bootstrapping the original data and the inter-quartile range (IQR).

**V44 duodenum**

|  | **p-value** | **AUC** | **OR** |
| --- | --- | --- | --- |
| *original*  *value* | *0.02* | *0.58* | *1.07* |
| **median*** | 0.02 | 0.61 | 1.08 |
| **IQR*** | 0.003- 0.15 | 0.55-0.68 | 1.05-1.10 |

**D_0.03_ duodenum**

|  | **p-value** | **AUC** | **OR** |
| --- | --- | --- | --- |
| *original*  *value* | *0.07* | *0.55* | *1.19* |
| **median*** | 0.09 | 0.61 | 1.18 |
| **IQR*** | 0.006-0.43 | 0.55-0.68 | 1.07-1.30 |

**V44 stomach**

|  | **p-value** | **AUC** | **OR** |
| --- | --- | --- | --- |
| *original*  *value* | *0.01* | *0.65* | *1.12* |
| **median*** | 0.03 | 0.66 | 1.12 |
| **IQR*** | 0.003-0.15 | 0.60-0.70 | 1.09-1.16 |

**D_0.03_ stomach**

|  | **p-value** | **AUC** | **OR** |
| --- | --- | --- | --- |
| *original*  *value* | *0.01* | *0.69* | *1.23* |
| **median*** | 0.004 | 0.69 | 1.23 |
| **IQR*** | 0.0003-0.03 | 0.64-0.75 | 1.17-1.31 |
